# Interplay of Opposing Effects of the WNT/β-Catenin Pathway and PPARγ and Implications for SARS-CoV2 Treatment

**DOI:** 10.3389/fimmu.2021.666693

**Published:** 2021-04-13

**Authors:** Alexandre Vallée, Yves Lecarpentier, Jean-Noël Vallée

**Affiliations:** ^1^ Department of Clinical Research and Innovation, Foch Hospital, Suresnes, France; ^2^ Centre de Recherche Clinique, Grand Hôpital de l’Est Francilien (GHEF), Meaux, France; ^3^ University Hospital Center (CHU) Amiens Picardie, University of Picardie Jules Verne (UPJV), Amiens, France; ^4^ Laboratory of Mathematics and Applications (LMA), Unité Mixte de Recherche (UMR) Centre National de la Recherche Scientifique (CNRS) 7348, University of Poitiers, Poitiers, France

**Keywords:** COVID-19, WNT/β-catenin pathway, PPARγ, ACE2, cytokine storm

## Abstract

The Coronavirus disease 2019 (COVID-19), caused by the novel coronavirus SARS-CoV-2 (severe acute respiratory syndrome coronavirus 2), has quickly reached pandemic proportions. Cytokine profiles observed in COVID-19 patients have revealed increased levels of IL-1β, IL-2, IL-6, and TNF-α and increased NF-κB pathway activity. Recent evidence has shown that the upregulation of the WNT/β-catenin pathway is associated with inflammation, resulting in a cytokine storm in ARDS (acute respire distress syndrome) and especially in COVID-19 patients. Several studies have shown that the WNT/β-catenin pathway interacts with PPARγ in an opposing interplay in numerous diseases. Furthermore, recent studies have highlighted the interesting role of PPARγ agonists as modulators of inflammatory and immunomodulatory drugs through the targeting of the cytokine storm in COVID-19 patients. SARS-CoV2 infection presents a decrease in the angiotensin-converting enzyme 2 (ACE2) associated with the upregulation of the WNT/β-catenin pathway. SARS-Cov2 may invade human organs besides the lungs through the expression of ACE2. Evidence has highlighted the fact that PPARγ agonists can increase ACE2 expression, suggesting a possible role for PPARγ agonists in the treatment of COVID-19. This review therefore focuses on the opposing interplay between the canonical WNT/β-catenin pathway and PPARγ in SARS-CoV2 infection and the potential beneficial role of PPARγ agonists in this context.

## Introduction

The Coronavirus disease 2019 (COVID-19), caused by the novel coronavirus SARS-CoV-2 (severe acute respiratory syndrome coronavirus 2), has quickly reached pandemic proportions. Like SARS-CoV, SARS-CoV-2 is a member of the Beta-coronavirus family. Although the majority of COVID-19 patients present mild to moderate clinical features (1, 2), some may develop severe pneumonia or suffer from the acute respiratory distress syndrome (ARDS) and multi-organ failure, leading to high death rates. Nevertheless, the pathophysiology of Corona Virus Disease-19 (COVID-19) remains unclear. Currently, in patients with life-threatening ARDS, there is growing evidence that virally-induced pro-inflammatory cytokines (such as Interleukin (IL)-6 and tumor necrosis factor-α (TNF-α)) enhance inflammation in the latter stages of this disease (3–5). Such findings are further corroborated by recent studies indicating that high levels of IL-6 are predictors of mortality (6). Cytokine profiles in COVID-19 patients have revealed increased levels of interleukin-1β (IL-1β), IL-2, IL-6 and tumor necrosis factor-alpha (TNFα) (7). TNF-α is one of the main activators of IL-6 expression and an increase in baseline plasma levels of IL-6 may predict that survival chances are poor (7). Moreover, an increase in the proportion of Th17 cells has been observed in COVID-19 patients, leading to the stimulation of IL-6 (8). Recent evidence has shown that the upregulation of the canonical WNT/β-catenin pathway is associated with inflammation and a cytokine storm in ARDS (9) and especially COVID-19 patients (10). Several studies have shown that, in numerous diseases (11–14), the WNT/β-catenin pathway interacts with PPARγ (peroxisome proliferator-activated receptor gamma) in an opposing interplay, with the effects of one opposing those of the other. Recent studies have also highlighted the possible role of PPARγ agonists as modulators of inflammatory and immunomodulatory drugs by targeting the cytokine storm in COVID-19 patients (15, 16). This review focuses on the opposing interplay between WNT/β-catenin and PPARγ in SARS-CoV-2 infection and the potential role of PPARγ agonists in this context.

## Inflammation and SARS-CoV-2 Infection

The severity of symptoms in SARS-CoV-2 infection depends on the viral infection and the host immune system. The COVID-19 cytokine profile of patients is closely associated with cytokine release, indicating macrophage activation, and an increase in the level of cytokines such as the TNFα, IL-6 and interferon-gamma (IFN-γ) (4).

The increased levels of these cytokines is a characteristic of ARDS, with a low level of oxygen in the blood and shortness of breath (17). The SARS-CoV-2 infection mainly damages the endothelial cells of the airway, alveoli, vascular system, and macrophages in the lungs. SARS-CoV-2 recruits the receptor of angiotensin-converting enzyme 2 (ACE2) for infection (18). The expression of the ACE2 receptor is decreased in the lungs in the SARS-CoV-2 infection, dysregulating the renin-angiotensin system, which damages the fluid and electrolyte balance, blood pressure levels, and increases the vascular permeability and inflammatory processes in the airway (19–21).

SARS-CoV recruits several immune-suppressive proteins thereby increasing the immune response (22). SARS-CoV enhances several structural and non-structural proteins acting as antagonists of interferon signaling. Stopping interferon signaling could be a response to: a) prevent the recognition of viral RNA *via* the pattern recognition receptor (PRR), b) decrease the synthesis of type I interferon protein by interrupting the toll-like receptor-1 (TLR-1) and the retinoic acid-inducible gene I (RIG-I), and c) increase the STAT pathway activity (23).

The SARS-CoV-2 virus causes massive damage to the infected epithelial and endothelial cells, with an excessive release of cytokines and chemokines (18). In SARS-CoV-2, stimulation of the caspase-1 enhances the production of pro-inflammatory cytokines such as IL-1β and IL-6 (24). These cytokines bind with other immune cells, including T-lymphocytes and monocytes (8, 25). Severe COVID-19 patients show increased levels of the granulocyte colony-stimulating factor (G-CSF), IL-2, IL-6, IL-10, monocyte chemo-attractant peptide (MCP)-1), macrophage inflammatory protein 1α (MIP1α) and TNF-α (26).

The nuclear factor-κB (NF-κB) pathway is one of the main inflammation processes. NF-κB is a hetero-dimeric transcription factor belonging to the Rel protein family. Under physiological conditions, RelA/p50, the heterodimer’s predominant form of the NF-κB pathway, is inactivated in the cytoplasm by the IkB protein (27). SARS-CoV infection leads to a release of pro-inflammatory cytokines and growth factors to activate the IkB Kinase (IKK), which phosphorylates and degrades the IkB protein through an ubiquitination mechanism (28).

The NF-κB pathway can modulate the expression of pro-inflammatory genes responsible for the cytokine storm. SARS-CoV-2 can induce the nuclear translocation of the NF-κB pathway to stimulate IL-6 expression (28). Numerous studies have shown that SARS-CoV (29–31), including SARS-Cov-2 (32), can activate the NF-κB pathway.

## The Canonical WNT/β-Catenin Pathway

The name WNT is derived from Wingless drosophila melanogaster and its mouse homolog Int. The canonical WNT/β-catenin pathway is involved in several mechanisms, controlling signaling, including embryogenesis, cell proliferation, migration and polarity, apoptosis, and organogenesis (33). Nevertheless, the WNT/β-catenin pathway can be altered in several pathological diseases, such as inflammation, metabolic, neurological and psychiatric disorders, fibrosis and cancer processes (34–42).

The WNT ligands belongs to the family of secreted lipid-modified glycoproteins (43). WNT ligands are produced by neurons and immune cells localized in the central nervous system (CNS) (44). WNT pathway dysfunction can affect numerous neurodegenerative pathologies (11, 45–48). The canonical WNT pathway comprises the β-catenin, T-cell factor and lymphoid enhancer factor (TCF/LEF). Cytoplasmic accumulation of β-catenin is modulated by the destruction complex AXIN, tumor suppressor adenomatous polyposis coli (APC), and glycogen synthase kinase-3 (GSK-3β). In the absence of WNT ligands, the destruction complex has a role in the phosphorylation of the cytoplasmic β-catenin and leads to its proteasomal destruction. However, when they are present, WNT ligands bind with Frizzled (FZL) and LDL receptor-related protein 5/6 (LRP 5/6) to interrupt the destruction complex and prevent β-catenin degradation into the proteasome. β-catenin translocates to the nucleus to interact with the TCF/LEF. This stimulates the WNT target genes (49–51).

Glycogen synthase kinase-3β (GSK-3β) is one of the major inhibitors of the WNT/β-catenin pathway (35, 36, 52–55). As an intracellular serine-threonine kinase, GSK-3β is a major negative controller of WNT signaling (56). GSK-3β is involved in the control of numerous kinds of pathophysiological pathways, including cell membrane signaling, cell polarity, and inflammation (57–59). GSK-3β interacts by downregulating the cytoplasmic β-catenin and stabilizing it to enhance its nuclear migration (60).

A positive interplay has been recently observed between the WNT/β-catenin pathway and inflammation, expressed by an activated NF-ϰB pathway (37). Over-stimulation of WNT/β-catenin leads to the enhancement of IϰB-α degradation and then NF-ϰB pathway transactivation (61). The WNT/β-catenin pathway increases COX expression, which then influences the inflammatory response (62). In addition, the NF-ϰB pathway downregulates GSK-3β and positively enhances β-catenin signaling (63, 64).

## WNT/β-Catenin Pathway and SARS-CoV-2 Infection

Several studies have shown that WNT ligands, secreted by immune cells, can control inflammatory response and immune cell modulation (65–68). Moreover, WNT ligands play major roles in tissue damage and repair (65). The WNT/β-catenin pathway is upregulated in severe sepsis-induced acute lung injury and sepsis mouse models (67, 69). The WNT pathway is damaged in sepsis or ARDS, and therefore plays a major role in fibrosis and inflammation (66, 70). In COVID-19 patients, the transforming growth factor (TGF-β) stimulates the WNT/β-catenin pathway, leading to an increased risk of pulmonary fibrosis (70) and pulmonary infection (10, 71) (**Table 1**). Several studies have shown that the TGF-β and WNT/β-catenin pathways upregulate each other in a positive feedback (54, 88).

**Table 1:** Mechanisms by which the WNT/β-catenin pathway is modulated and the possible roles of PPARγ agonists in treating SARS-CoV-2 infection.

Target	Expression	Co-modulator	Disease complications	Model	References
WNT/β-catenin	Increase	TGF-β	Pulmonary fibrosis	COVID-19 patients	(65)
WNT/β-catenin	Increase	TGF-β	Pulmonary infection	COVID-19 patients	(10, 67)
serum IL-6	Increase	–	–	COVID-19 patients	(72)
IL-10, TGF-β	Increase	PAI-1	Pulmonary fibrosis	COVID-19 patients	(73)
TGF-β	Increase	–	ECM dysregulation	COVID-19 patients	(74, 75)
TGF-β	Increase	PAI-1 and collagen I	Lung fibrosis	SARS-coronavirus patients	(76)
ACE2	Decrease	Spike (S) viral protein	Fibrosis, endothelial dysfunction, increased inflammation, oxidative stress	COVID-19 patients	(77–80)
ACE2	Increase	pioglitazone	–	Animal models	(81)
ACE2	Increase	pioglitazone	–	Hypothesis research in COVID-19 patients	(82)
NF-κB	Decrease	Pioglitazone	–	COVID-19 patients	(15, 83)
Cytokines storm	Decrease	PPARγ agonists	–	COVID-19 patients	(84–86)
SARS-CoV-2 RNA synthesis and replication	Decrease	Pioglitazone (as 3CL-Pro inhibitor)	–	Hypothesis research in COVID-19 patients	(87)

ACE2, angiotensin-converting enzyme 2; COVID-19, Coronavirus disease 2019; Il-6, Interleukin-6; NF-κB, Nuclear factor-κB pathway; PPARγ, peroxisome proliferator-activated receptor gamma; SARS-CoV, severe acute respiratory syndrome coronavirus; TGF-β, transforming growth factor-beta; TNF-α, tumor necrosis factor-α.

The TGF-β pathway is one of the main signaling pathways involved in fibrosis through the enhancement of EMT and fibroblast differentiation (89). Several inflammatory cytokines have a positive relationship with the TGF-β pathway (89). Interactions between the TGF-β pathway and Smad pathway are involved in pulmonary fibrosis (90). The TGF-β/Smad pathway has been shown to be a promotor of PAI-1 synthesis in SARS-CoV (91). Moreover, cytokines can activate the JAK/STAT pathway (92) to dysregulate cellular homeostasis, proliferation and apoptosis (93). IL-6 can activate the JAK/STAT pathway in T helper cells (4, 94) to induce several biological functions, such as immune regulation, lymphocyte differentiation and oxidative stress (72, 95). The increase in IL-6 observed in severe COVID-19 patients is associated with significantly lower survival rates (6, 96). COVID-19 patients present both dysregulated JAK/STAT pathway (97) and important role of TGF-β/Smad pathway (98).

In severe COVID-19 patients, serum IL-6 is significantly greater than in non COVID-19 subjects (99). The excessive production of inflammatory cytokines in the lungs of COVID-19 patients is associated with the increase in macrophage activation (100). In a mouse model of systemic inflammation, PAI-1 is involved in the regulation of host inflammatory responses through Toll- like Receptor-4 (TLR4)-mediated macrophage activation (101). Activation of the NF-κB pathway results in stimulating the TGF-β pathway in a vicious loop (73) and in concordance with PAI-1 (74). Thus, PAI-1 seems to be partly responsible for the excessive production of cytokines by macrophages in severe COVID-19 patients (75). PAI-1 expression is associated with increased IL-10 and an activated TGF-β pathway (102). Thus, the activated TGF-β pathway observed in COVID-19 patients may drive the pulmonary fibrosis process (102). In COVID-19 patients, ECM dysregulation could be one of the sources of stimulation of the TGF-β pathway (76, 103) (**Table 1**). This stimulation is responsible for the activation of integrin αvβ6 and thrombospondin induced by the STAT pathway (76, 104). In COVID-19 patients, a vicious loop is involved between the TGF-β pathway, the STAT pathway and PAI-1 (75). Furthermore, the targets involved in fibrosis, such as collagens, proteoglycans, integrins, the connective tissue growth factor, and matrix metalloproteinases (MMPs) are activated by the TGF-β pathway (105). SARS-CoV proteins may enhance the TGF-β-induced expression of PAI-1 and collagen I to induce lung fibrosis (106).

## PPARγ

PPARs (peroxisome proliferator-activated receptors) are ligand-activated transcription factors that bind PPREs (PPAR-response elements). In the nucleus, PPARs form a heterodimer with the retinoid X receptor (RXR) (107). They are composed of a ligand-binding domain that interacts with a DNA-binding domain to modulate it (108). PPARs are involved in numerous pathophysiological processes, such as cell differentiation, protein metabolism, lipid metabolism, carcinogenesis (109, 110), adipocyte differentiation, insulin sensitivity and inflammation (111, 112). PPARs are subdivided into three isoforms: PPARα, PPARγ and PPARβ (113). PPARγ is highly expressed in adipose tissue and macrophages (114). PPARγ ligands can be synthetic or natural. PPARγ ligands have hypoglycemic and hypocholesterolemic roles. PPARγ agonists such as thiazolidinediones (TZDs) have been used to treat type 2 diabetes patients (115) and to decrease inflammatory activity (115). Natural ligands include prostaglandins and unsaturated fatty acids (116). Natural ligands include prostaglandins and unsaturated fatty acids. Moreover, PPARγ ligands, such as thiazolidinediones, can directly decrease inflammatory activity (12), fibrosis processes (117) and lung inflammation (118). In adipocyte mitochondria, pioglitazone (30–45 mg/day for three months) can reduce the expression of cytokines, including IL-6 and TNFα in humans (119). In patients with impaired glucose tolerance, four months (45 mg/day) of treatment with pioglitazone is associated with a reduction of monocyte IL-1, IL-6, IL-8 and lymphocyte IL-2, IL-6 and IL-8 (120). Pioglitazone has also shown a potential for decreasing ferritin in a rat model of angiotensin II-induced hypertension (121). Moreover, pioglitazone can decrease the secretion of pro-inflammatory cytokines (IL-1b, IL-6, and IL-8) and increase the anti-inflammatory ones (e.g. IL-4 and IL-10) in astrocytes stimulated by lipopolysaccharide (122). Pioglitazone (treatment for 7 days) could decrease TNFα and IL-6 mRNA expression in the peritoneal lavage fluid (15, 123). Furthermore, pioglitazone is a well-known inhibitor of lung inflammation and fibrosis (118), through normalization of the expression of TNF-α (124). Pioglitazone and rosiglitazone use can reduce both the increase in inflammatory markers and the decrease in the glutamate transporter (GLT-1) expression, in a primary mixed culture of astrocytes and microglia caused by exposure to *in vitro* viral proteins (HIVADA gp120) and *in vivo* models (125). Pioglitazone can decrease the neuro-inflammation and maintain mitochondrial respiration (126). The use of pioglitazone has also produced encouraging results in the form of decreasing CRP and IL-6 levels (127). In animal studies, pioglitazone has been shown to decrease mortality from sepsis and lung injury by reducing inflammatory cytokine production in omental tissue (123).

## Opposing Interplay Between the WNT/β-Catenin Pathway and PPARγ

Several studies have shown that the canonical WNT/β-catenin pathway and PPARγ act in an opposing manner in numerous disorders, including cancers, neurodegenerative diseases, psychiatric disorders and fibrosis processes (47, 117, 128). In many diseases, PPARγ expression is downregulated by β-catenin signaling over-activation (12, 13, 48, 129–131). PPARγ agonists are considered to offer promising treatment through this crosstalk process (13, 132, 133). Indeed, PPARγ is considered to be a negative β-catenin target gene (134, 135). The WNT/β-catenin pathway and PPARγ interact through a TCF/LEF domain of β-catenin and a catenin-binding domain within PPARγ (77, 136). Through this link, a decrease in WNT/β-catenin pathway activity enhances the activation of PPARγ (78), while PPARγ over-expression inhibits β-catenin signaling (79).

## Opposing Interplay Between the WNT/β-Catenin Pathway and PPARγ in SARS-CoV-2 Infection: The ACE2 Hypothesis

SARS-CoV-2 uses the angiotensin-converting enzyme 2 (ACE2) as a main cell receptor to infect humans (80, 137–139) (**Table 1**). ACE2 plays a leading role in the regulation of cardiovascular and renal functions (140) and has also been shown to have a major role in SARS-CoV-2 infection (80, 138). SARS-CoV-2 may invade human organs besides the lungs through the expression of ACE2 (141). Recent findings have revealed that ACE2 is downregulated in SARS-CoV-2-infected lung tissue (142). Evidence from studies has shown that SARS-CoV-2 gains direct access to cells through ACE2 receptors (80, 143), as happens with SARS-CoV (144). SARS-CoV-2 infection leads to the downregulation of the expression of ACE2 by binding with the spike (S) viral protein – 1273 amino acid long protein (145). The pivotal role of ACE2 is its degradation of angiotensin II into angiotensin_1-7_ (146). This degradation mechanism is blocked by a selective ACE2 inhibitor, such as MLN-4760 (147). A recent study focusing on SARS-CoV-2 has shown that angiotensin II accumulation leads to fibrosis, endothelial dysfunction, increased inflammation and oxidative stress (81). Moreover, angiotensin II is associated with macrophage activation and both IL-6 and TNF-α overexpression (148). Furthermore, the deficiency in ACE2 could exacerbate outcomes in COVID-19 patients (148). In COVID-19 patients, ACE2 expression is inversely associated with the WNT/β-catenin and TGF-β pathways (141). ACE2 presents a positive association with PD-L1, a predictive marker for active response to immune inhibitors (142). The stimulation of ACE2 allows it to play a major protective role in the treatment of hypertension, heart disease, cancer and COVID-19 (141), which are all disorders that show an upregulation of the WNT/β-catenin pathway. Rats with renal ischemia/reperfusion-induced injury treated by pioglitazone have shown a downregulated WNT/β-catenin pathway and increased ACE2 expression (82). Even though very few studies have so far highlighted the possible role of PPARγ agonists in treating COVID-19 patients, rosiglitazone has been shown to increase ACE2 expression in animal models (81) and it could also potentially be used in diabetic patients with COVID-19 (85).

## PPARγ Agonists in SARS-CoV-2 Infection

To date, few studies have focused on the potentially interesting link between PPARγ agonists and the development of COVID-19. The role of these agonists in SARS-CoV-2 infection therefore remains hypothetical (15, 16) (**Table 1**). Currently, no clinical and randomized trials have been investigated. However, in a recent research review article it was hypothesized that pioglitazone could play a role in attenuating lung injury in COVID-19 patients (15). Pioglitazone is another available thiazolidinedione that may inhibit the activation of NF-kB and MAPK pathways by reducing the expression of CARD9 in COVID-19 patients (15, 86). Several reports have indicated that PPARγ agonists could be candidates for modulating the cytokine storm in the COVID-19 disease (87, 149, 150). A possible therapeutic role for pioglitazone has been identified with respect to the SARS-CoV-2 infection (16). Pioglitazone can act as a 3CL-Pro inhibitor to downregulate SARS-CoV-2 RNA synthesis and replication (151). More specifically, PPARγ agonists can decrease the secretion of several pro-inflammatory cytokines, including TNF-α, IL-1, and IL-6, in both the monocytes and macrophages (152).

Recent studies have shown that numerous COVID-19 patients present hypertension and diabetes, whereas few patients present chronic obstructive-pulmonary diseases (153, 154). Moreover, a recent meta-analysis showed that hypertension and diabetes were highly associated with comorbidities in COVID-19 patients (155). One of the major roles of PPARγ agonists is to decrease TNF-α expression, the proportion of Th17 cells and NF-κB activity in order to repress inflammation (12). Numerous inflammatory cytokines, chemokines, or intracellular pathways, such as TNF-α and IL-6, can downregulate PPARγ expression, whereas in adipocytes, adiponectin increases PPARγ expression and then downregulates the LPS-induced NF-ϰB expression and IL-6 production (156). Pioglitazone suppresses inflammation by reducing TNF-α and MCP-1 expression, two important mediators of inflammation (157). However, the use of PPARγ agonists may have some side effects, even though newer molecules now have fewer disadvantages. The use of PPARγ agonists may therefore increase cardiovascular events, despite numerous studies showing no significant increase in side effects (14).

## Conclusion

In the rapidly evolving situation surrounding the COVID-19 pandemic, it is essential to better understand the different pathways involved in the disease. In the SARS-CoV-2 infection, the canonical WNT/β-catenin pathway seems to be upregulated in association with the TGF-β and STAT pathways, whereas both ACE2 and PPARγ expression is downregulated, coupled with an increased number of pro-inflammatory markers. Since increased WNT/β-catenin pathway activity is associated with the increase of immune signaling and fibrosis processes, the inhibition of this pathway could result in the negative modulation of the SARS-CoV-2 infection. PPARγ agonists provide inexpensive treatments that are commonly used around the globe. By directly targeting inflammation, ACE2 and the WNT/β-catenin pathway, PPARγ agonists may well be prospective candidates for delivering SARS-CoV-2 therapy in clinical settings.

## Author Contributions

All authors listed have made substantial, direct, and intellectual contributions to the work and approved it for publication.

## Conflict of Interest

The authors declare that the research was conducted in the absence of any commercial or financial relationships that could be construed as a potential conflict of interest.
